# A comparison of traditional diarrhoea measurement methods with microbiological and biochemical indicators: A cross-sectional observational study in the Cox's Bazar displaced persons camp

**DOI:** 10.1016/j.eclinm.2021.101205

**Published:** 2021-11-20

**Authors:** Ryan Rego, Samuel Watson, Mohammad Atique Ul Alam, Syed Asif Abdullah, Mohammad Yunus, Imam Taskin Alam, A.S.M.Homuan Kabir Chowdhury, S.M.Arefeen Haider, ASG Faruque, Azharul Islam Khan, Timothy Hofer, Paramjit Gill, Mohammad Sirajul Islam, Richard Lilford

**Affiliations:** a Center for Global Health Equity, University of Michigan at Ann Arbor, USA; b Center for Global Health, University of Warwick, UK; c Institute for Applied Health Research, University of Birmingham, UK; d International Center for Diarrhoeal Disease Research, Bangladesh

**Keywords:** Diarrhoea, Diagnostics, Refugee, Enteric, Infection, Epidemiology

## Abstract

**Background:**

Water, Sanitation, and Hygiene (WASH) systems aim to reduce the spread of enteric pathogens, particularly amongst children under five years old. The most common primary outcome of WASH trials is carer-reported diarrhoea. We evaluate different diarrhoea survey instruments as proxy markers of enteric pathogen presence in stool.

**Methods:**

We recruited 800 community-based participants from the Cox's Bazar Displaced Person's Camp in Bangladesh, split evenly between the rainy (July/August 2020) and dry (November/December 2020) periods. Participants were randomized evenly into either a standard survey asking carers if their child under five years old has had diarrhoea in the past fortnight, or a pictorial survey asking carers to pick from a pictorial chart which stools their child under five years old has had in the past fortnight. We collected stools from a random sub-sample of 120. Stools were examined visually, and tested for proteins associated with enteric infection and 16 enteric pathogens. We calculated sensitivities and specificities for each survey type, visual examination, and proteins with respect to enteric pathogen presence.

**Findings:**

The sensitivity of the standard survey for enteric pathogen presence was 0.49[95%CI:0.32,0.66] and the specificity was 0.65[0.41,0.85]. Similar sensitivities and specificities were observed for pictorial survey, visual inspection, and proteins.

**Interpretation:**

While diarrhoea is an important sign in clinical practice it appears that it is a poor proxy for enteric pathogen presence in stool in epidemiological surveys. When enteric infection is of interest, this should be measured directly.

**Funding:**

The project was funded by the National Institutes for Health Research Global Health Research Unit on Improving Health in Slums (16/136/87) and by the University of Warwick.

**Research in context**

**Evidence before this study**

Recent large scale trials of water, sanitation, and hygiene (WASH) interventions have shown limited to no intervention effectiveness, likely due to these trials generally using carer-reported diarrhoea obtained from community surveys as their primary outcome. The unwritten assumption is that diarrhoea obtained from carer-reports is a good proxy marker of enteric pathogen presence in stool. We searched Medline, Embase, and PubMed for studies published between 2000 and 2018 that evaluated survey-based reports of diarrhoea as a proxy marker of enteric pathogen presence in stool, and found no such studies.

**Added value of this study**

We carried out an observational study to evaluate four different methods of measuring diarrhoea as proxy markers of enteric pathogen presence in stool, including the standard DHS/UNICEF recommended method, and three alternatives. The standard method was very poorly correlated with pathogen presence with a sensitivity and specificity of about 0.5, meaning that it was about as discriminating as the toss of a coin. Moreover, we found that the alternative methods, surveys using pictorial charts, direct observation of stool and inflammatory markers in stool, were no better.

**Implications of all the available evidence**

The presence or absence of diarrhoea, regardless of method of measurement, is not a good proxy marker of enteric pathogen presence in stool. Insofar as WASH interventions aim to reduce the ‘burden’ of infection and carriage of enteric pathogens in children under five years old, community surveys on diarrhoea, even those involving direct observation of stool, are poor signals for effectiveness. While clinical outcomes are usually preferable to laboratory measurements in clinical epidemiology, in the particular case of WASH interventions, our observations suggest that laboratory observations may be a preferable primary outcome.

Alt-text: Unlabelled box

## 1 Introduction

Diarrhoea, often caused by enteric pathogens, is a main cause of death in children under five in low and middle income countries (LMICs) [1,2]. Of additional concern is stunted linear growth and malnutrition. Considerable efforts have been made to tackle these problems by reducing the exposure of under-fives to enteric pathogens in faeces through large-scale water, sanitation, and hygiene (WASH) interventions. WASH interventions have been evaluated in observational studies and randomised trials, including three recent large-scale trials [3], [4], [5], [6], [7], [8], [9]. These recent trials mostly found limited evidence for the effectiveness of WASH interventions in reducing under-five diarrhoea and stunted linear growth [3,4,8]. Since there is overwhelming evidence of water and environmental contamination as the primary sources of enteric pathogens which are in faeces, it is widely argued that the WASH interventions in these studies were insufficient; either in reducing exposure to enteric pathogens in faeces or in providing services to enough of the population enough of the time [10]. In this paper we examine another factor that may explain some of the disappointing results of WASH evaluations, the method through which WASH interventions are evaluated.

The standard primary outcome in WASH evaluations is carer-reported diarrhoea from community surveys [11]. Clinic visits for diarrhoea are seldom used, as not only 2% of diarrhoea cases report to the clinic, often the most severe cases [11], [12], [13]. The use of community surveys rests on an important assumption, that there is a sufficiently strong correlation between survey-based diarrhoea and the presence of enteric organisms in the gut.

Several factors together cast some doubt on the adequacy of survey-based diarrhoea rates as a proxy for the presence of enteric pathogens in stool. First, survey reported diarrhoea rates are highly subjective and are heavily influenced by how the questionnaire is framed [11]. Second, diarrhoea may have causes other than enteric infection, which WASH interventions will not affect [16], [17], [18], [19]. Last, asymptomatic children may still carry and shed enteric pathogens which can cause morbidity in others. WASH interventions are designed to reduce both enteric infection and asymptomatic carriage, reducing the community's total pathogen load. Evaluations must therefore examine the total pathogen load in the community. However, given the aforementioned factors together, the use of diarrhoea as a proxy marker of pathogen presence may result in misclassification.

We explore how well survey results classify the presence of enteric pathogens in stool. This question is of practical importance as the cost of microbiological analysis of stool has dropped considerably in recent years [14]. Hence, microbiological sampling is now a potential epidemiological, as well as clinical, tool for use in intervention trials, surveillance activities, modelling studies, and economic studies.

In the Cox's Bazar Forcibly Displaced Myanmar Nationals Camp we evaluated four methods of measuring diarrhoea as proxy markers of enteric pathogen presence in stool: the standard survey question-based method and three alternatives. The standard method, recommended by UNICEF and the Demographic and Health Surveys (DHS), is used in most evaluations of WASH interventions: asking a carers if their child has had diarrhoea in the past 14 days [15]. The alternatives are: 1) pictorial surveys, asking carers to pick from a pictorial stool chart which stools their child has had in the past 14 days [16]; 2) visual confirmation, where trained researchers visually examine the stool [17]; and 3) analysis of the stool for two proteins associated with diarrhoea [18,19]. We calculated the agreement between standard verbal surveys, pictorial surveys, and visual stool analysis.

## 2 Methods

### 2.1 Study design

**Fig. 1:**
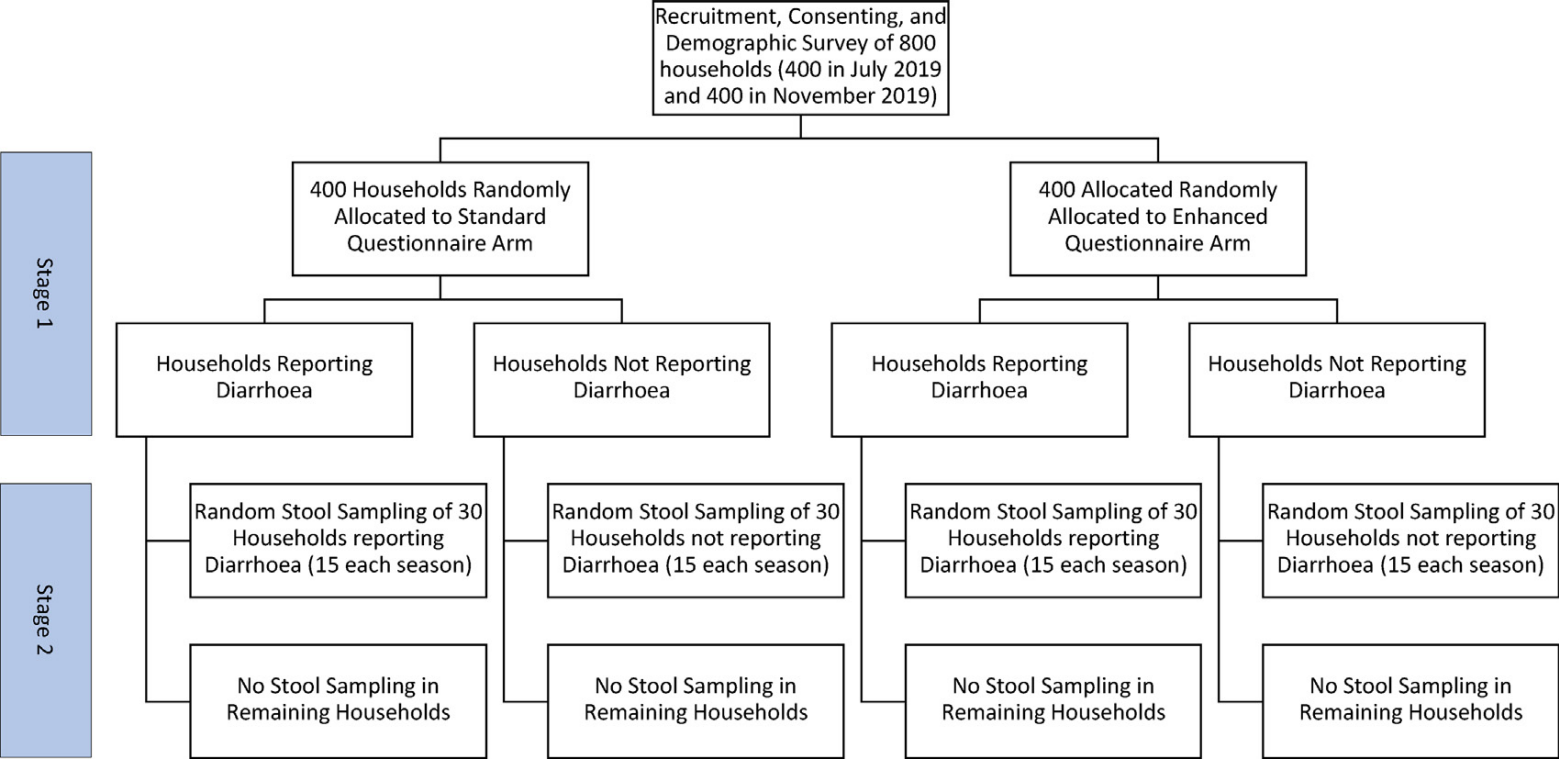
Study design.

Fig. 1 presents the study flow chart. We collected cross-sectional data in the wet season and the dry season (July/August 2020, and November/December 2020). Given limited resources for comprehensive laboratory analysis we ensured that we would have adequate numbers of people reporting both diarrhoea and no diarrhoea by conducting the study in two stages. Stage one consisted of a survey of 800 participants, who were randomly assigned into a standard questionnaire arm or a pictorial survey arm in a 1:1 ratio through simple random sampling. The second stage was collection of stools from a subset of participants. We aimed to collect 120 stools, split evenly by season, survey arm and by diarrhoea report status. The randomisation strategy for arm allocation and stool collection is described in Appendix 1.

### 2.2 Study setting

Cox's Bazar is home to over one million Forcibly Displaced Myanmar Nationals (FDMNs). This study took place in the Leda Makeshift Camp. The first round of data collection took place between July and August 2019, the rainy and warm season (average temperature 32 °C with 25–27 rainy days per month); and the second round of data collection took place between November and December 2019, the dry and cooler season (average temperature 28 °C with 2–3 rainy days per month) [20].

### 2.3 Recruitment

Each day, we drew a sample from a map of all households in the sampling area that had not previously been visited (Appendix 1). On arriving at a household, we checked for the presence of an adult. If no adult was present, we returned daily until one was. We then asked if the household had a child under the age of five. If not, we moved on to the next closest household. On finding a household with an under-five a full explanation of the study was given. If the adult respondent wished to participate, the participant was given a participant information letter which was explained to them verbally, and asked to sign a consent form which was read to them if necessary. We then evaluated the household against the inclusion and exclusion criteria: having at least one under-five, the respondent being over 18, and the household not expecting to relocate in the next six months (as to not preclude the possibility of follow up). If a household had more than one under-five, we selected the oldest as the participant.

## 3 Test methods

### 3.1 Stage one: surveys

We first administered a demographic survey, using questions from the Demographic and Health Surveys [21]. This survey also included questions on key risk factors for diarrhoea, such as source of water and type of latrine used. Subsequently, we administered either the standard or pictorial diarrhoea questionnaire. All surveys can be found in Appendix 2.

### 3.2 The standard questionnaire

Participants in the standard arm received the UNICEF/DHS recommended questionnaire which enquired whether the oldest child under-five in their care has ‘had three or more loose or watery stools any day in the past two weeks’. We also asked if their under-five has had blood in their stool in the past 14 days and, if so, if and where they sought care [15].

### 3.3 The pictorial questionnaire

Participants in the pictorial arm were shown the Amsterdam Stool Chart (Appendix 3) and asked to select all the pictures resembling the stools observed from their under-five over the past 14 days [22]. If loose or watery stools were reported, we asked how many times the child had this stool type on the worst day and how many days this stool type lasted. We then, regardless of diarrhoea, asked about symptoms of other water-washed diseases in the under-five over the last 14 days: fever, blood in stool, vomiting, eating problems, rashes, and eye problems [23]. If respondents answered yes to any of these, we asked if and where care was accessed.

### 3.4 Stage two: stool sampling

We asked a random subset of carers to provide stool samples from their oldest under-five. We gave those who agreed a large container and instructed them that their oldest under-five should defecate into it, and that we would return the next morning for collection. If the household did not provide stool the next morning, we asked why and if they would be able to provide a sample the next day. If they did not agree, the household was marked as lost to follow up. Further, if the household was not reachable after two days, or did not provide a stool sample by Thursday of the week (the last day before the weekend), they were marked as lost to follow up. Replacements were sought through the collection of additional samples from participants later in the study.

When a household provided a stool sample, we visually examined its consistency. We then transferred the stool into a specimen container and placed it on ice. Within 8 h, the stool was frozen to −20 °C and later transported to Dhaka for laboratory analysis. Stool was tested in the laboratory for 16 endemic pathogens and two proteins associated with diarrhoea; calprotectin and lactoferrin (Appendix 4).

### 3.5 Analysis

Calculations used to estimate our sample size can be found in Appendix 5. Using the *ci means* command in Stata, we summarised the survey data by calculating the means and 95% confidence intervals of key demographic variables, selected risk factors for diarrhoea, and measurements of diarrhoea: broken down by season, data collection arm, and stool collection status. We defined ‘having diarrhoea’ for each measure of diarrhoea as: (1) answering yes to the standard survey; (2) stating diarrhoea types A or B on the pictorial survey; (3) having faecal calprotectin level over 50 µg/g and/or a faecal lactoferrin level over 7.25 µg/g for protein measurements [18,19]; or (4) stool visually being loose or watery for visual confirmation. We also summarized results of the stool tests for enteric pathogens, broken down by survey type, season, and diarrhoea status (through each measure) – with 95% confidence intervals calculated. Rates of each enteric pathogen being present in all stools within seasons were calculated by weighting by applying frequency rates per data collection arm and diarrhoea status (Appendix 6).

Our main outcome was the performance of the four measures of diarrhoea as proxy markers of enteric pathogens being present in stool, in terms of sensitivity and specificity. The ‘reference standard’ used was PCR detected pathogens, compared against the each of the proxy measures. These diagnostic performance indicators were calculated for pathogen presence as a whole (the reference standard being PCR detecting any pathogen in stool), and for specific categories of pathogens (e.g. bacterial pathogens in stool).

Our secondary outcome measure was the agreement between the different measures for diarrhoea. We calculated this through computation of kappa values, along with expected and observed agreements.

### 3.6 Ethics

Ethics was granted by the University of Warwick BSREC in the United Kingdom (REGO-2019–2345) and by the ICDDR,B Ethical Review Committee in Bangladesh (PR-19,027). The study was also approved by the Refugee Relief and Repatriation Commission (Letter 789). This study and its protocol was prospectively registered on ISRCTN and updated as needed (ISRCTN41564300).

### 3.7 Role of the funding source

The funder had no role in study design, implementation, or interpretation.

## 4 Results

### 4.1 Recruitment

**Fig. 2:**
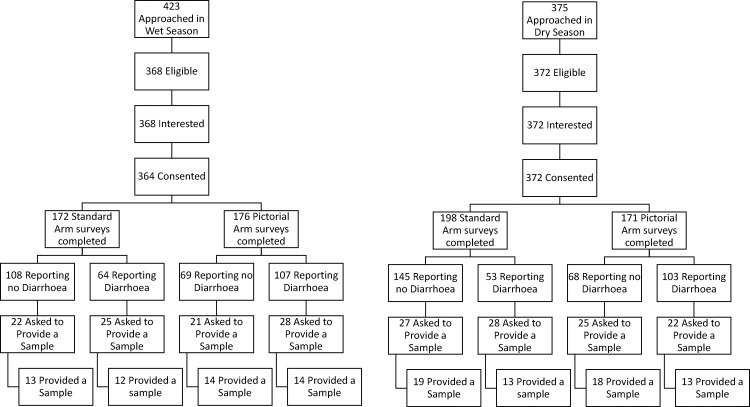
Recruitment flow chart.

Fig. 2 shows the participant disbursement. In the wet season we approached 423 households of which 368 were eligible. All but four of these eligible households consented to take part, of which 348 completed the study. The sixteen non-completers either withdrew consent during data collection or had to leave during data collection (e.g. to collect water). We asked 96 of the completing households to provide a stool sample and 56 did.

In the dry season, we approached 375 households of which 372 were eligible. All eligible households consented to take part, of which 369 completed the study. The non-completers either withdrew consent during data collection or had to leave for another reason. We asked 102 of the completing households to provide stool samples and 63 did.

Due to civil unrest in the wet season and Cyclone Bulbul in the dry season, we concluded data collection early. This resulted in a shortfall of 52 surveys (348/400) and 4 stool samples (56/60) in the wet season, and 31 surveys (369/400) in the dry season . Sixty-three planned stool samples were collected in the dry season.

Baseline Demographic Data and Outcome data

### 4.2 Demographic variables

Appendix 7 reports the demographics and selected diarrhoea risk factors, broken down by arm, season, and diarrhoea report.

### 4.3 Diarrhoea rates and inflammatory markers in stool

Table 1 reports the diarrhoea rates estimated from each proxy marker. Across both seasons, 32% [95%CI:27,37%] in the standard survey arm reported diarrhoea, compared to 60% [55,65] in the pictorial survey arm. 49% [40,59] of stools were visually loose or watery, and 77% [69,85] of stools had elevated levels of calprotectin (>7.25 µg/g) and/or lactoferrin (>50 µg/g).

**Table 1:** Diarrhoea rates obtained through each proxy measure.

	**Wet Season**	**Dry Season**	**Overall**
**Standard Survey**	37.2 [29.9, 44.5]	30.0 [20.6, 33.4]	31.9 [27.0, 36.7]
**Pictorial Survey**	60.8 [53.5, 68.1]	59.6 [52.2, 67.1]	60.2 [55.1, 65.4]
**Visual Analysis**	48.0 [33.7, 62.3]	51.5 [39.1, 63.9]	49.6 [40.4, 58.8]
**Protein Marker**	100.0 [93.5, 100]	57.6 [45.3, 69.8]	76.9 [69.2, 84.5]

Percentages and 95% Confidence Intervals of Each Measure of Diarrhoea Across Seasons:*% [95%CI]*.

### 4.4 Pathogens present in stool

Table 2 reports the rates of presence of the enteric pathogens in stool – broken down by season, survey arm, and reported diarrhoea status. Overall, 65% [51,78] of stools in the wet season had at least one enteric pathogen present versus 79% [69,90] in the dry period. Bacterial pathogens were present more often in the wet season (where 42% [28,55] of stools had at least one bacterial pathogen) than the dry season (19% [9,29]). Viral pathogens were present more often in the dry season (70% [58,82]) than in the wet season (38% [25,51]).

**Table 2:** Rates of enteric pathogens present in stool, broken down by season, survey arm, and reported stool status.

**Wet Season*%[95%CI]***						
		**Standard Survey**		**Pictorial Survey**		**Overall (*n*** **=** **56)**
**Type**	**Species**	**Healthy (*n*** **=** **13)**	**Diarrhoea (*n*** **=** **12)**	**Healthy (*n*** **=** **14)**	**Diarrhoea (*n*** **=** **17)**	
**Bacteria**	**Enteropathogenic Escherichia Coli**	23.1 [0.0, 49.5]	33.3 [2.0, 64.6]	0.0 [0.0, 3.3]	11.7 [0.0, 28.8]	15.7 [5.7, 25.7]
	**Enterotoxigenic E. Coli**	23.1 [0.0, 49.5]	16.7 [0.0, 41.3]	35.7 [7.0, 64.4]	17.6 [0.0, 37.8]	23.0 [11.5, 34.6]
	**Shiga Toxin Producing E. Coli**	0.0 [0.0, 3.3]	0.0 [0.0, 3.4]	0.0 [0.0, 3.3]	0.0 [0.0, 3.1]	0 [0.0, 2.7]
	**Campylobacter**	0.0 [0.0, 3.3]	0.0 [0.0, 3.4]	7.1 [0.0, 22.6]	11.7 [0.0, 28.8]	5.6 [0.0, 11.9]
	**Salmonella**	0.0 [0.0, 3.3]	16.7 [0.0, 41.4]	14.2 [0.0, 35.2]	0.0 [0.0, 3.1]	5.7 [0.0, 11,5]
	**Shigella**	0.0 [0.0, 3.3]	0.0 [0.0, 3.4]	7.1 [0.0, 22.6]	0.0 [0.0, 3.1]	1.6 [0.0, 4.7]
	**Cholera**	0.0 [0.0, 3.3]	0.0 [0.0, 3.4]	0.0 [0.0, 3.3]	0.0 [0.0, 3.1]	0 [0.0, 2.7]
	**Any Bacteria**	**46.2 [14.8, 77.5]**	**50.0 [16.8, 83.1]**	**50.0 [20.0, 80.0]**	**29.4 [5.3, 53.6]**	**41.9 [28.4, 55.4]**
**Protozoa**	**Entamoeba histolytica**	0.0 [0.0, 3.3]	0.0 [0.0, 3.4]	0.0 [0.0, 3.3]	0.0 [0.0, 3.1]	0 [0.0, 2.7]
	**Cryptosporidium**	0.0 [0.0, 3.3]	0.0 [0.0, 3.4]	0.0 [0.0, 3.3]	0.0 [0.0, 3.1]	0 [0.0, 2.7]
	**Giardia**	0.0 [0.0, 3.3]	8.3 [0.0, 26.7]	14.2 [0.0, 35.3]	0.0 [0.0, 3.1]	4.4 [0.0, 9.6]
	**Any Protozoa**	**0.0 [0.0, 3.3]**	**8.3 [0.0, 26.7]**	**14.2 [0.0, 35.3]**	**0.0 [0.0, 3.1]**	**458 [0.0, 9.6]**
**Viruses**	**Rotavirus**	0.0 [0.0, 3.3]	0.0 [0.0, 3.4]	0.0 [0.0, 3.3]	0.0 [0.0, 3.1]	0 [0.0, 2.7]
	**Sapovirus**	0.0 [0.0, 3.3]	8.3 [0.0, 26.7]	7.1 [0.0, 22.6]	5.9 [0.0, 18.4]	4.9 [0.0, 10.5]
	**Hepatitis E**	0.0 [0.0, 3.3]	0.0 [0.0, 3.4]	0.0 [0.0, 3.3]	0.0 [0.0, 3.1]	0 [0.0, 2.7]
	**Adenovirus**	38.5 [7.9, 69.1]	16.7 [0.0, 41.4]	21.4 [0.0, 46.0]	11.8 [0.0, 29.8]	22.2 [10.7, 33.8]
	**Astrovirus**	0.0 [0.0, 3.3]	0.0 [0.0, 3.4]	0.0 [0.0, 3.3]	0.0 [0.0, 3.1]	0 [0.0, 2.7]
	**Norovirus**	15.3 [0.0, 38.1]	25 [0.0, 53.7]	28.6 [1.5, 55.6]	5.9 [0.0, 18.4]	16.6 [6.6, 26.5]
	**Any Virus**	**38.5 [7.9, 69.1]**	**41.6 [8.9, 74.4]**	**57.1 [27.5, 86.8]**	**23.5 [1.0, 46.0]**	**38.0 [24.7, 51.2]**
**Any Pathogen**		**69.2 [40.2, 98.3]**	**75.0 [46.3, 100]**	**78.5 [54.0, 100]**	**47.1 [20.6, 73.5]**	**64.6 [51.4, 77.9]**
**Dry Season*%[95%CI]***						
		**Standard Survey**		**Pictorial Survey**		**Overall (*n*** **=** **63)**
**Type**	**Species**	**Healthy (*n*** **=** **19)**	**Diarrhoea (*n*** **=** **13)**	**Healthy (*n*** **=** **18)**	**Diarrhoea (*n*** **=** **13)**	
**Bacteria**	**Enteropathogenic E. Coli**	0.0 [0.0, 3.0]	0.0 [0.0, 3.3]	16.7 [0, 35.7]	0.0 [0.0, 3.3]	4.6 [0.0, 9.7]
	**Enterotoxigenic E. Coli**	15.8 [0.0, 33.8]	7.7 [0.0, 24.5]	11.1 [0, 27.1]	7.7 [0.0, 24.5]	11.5 [3.2, 19.8]
	**Shiga Toxin Producing E. Coli**	0.0 [0.0, 3.0]	7.7 [0.0, 24.5]	5.6 [0.9, 17.3]	7.7 [0.0, 24.5]	4.4 [0.0, 9.4]
	**Campylobacter**	0.0 [0.0, 3.0]	15.3 [0.0, 38.1]	5.6 [0.0, 17.3]	7.7 [0.0, 24.5]	5.6 [0.0, 11.2]
	**Salmonella**	0.0 [0.0, 3.0]	7.7 [0.0, 24.5]	0 [0.0,3.1]	0.0 [0.0, 3.3]	1.2 [0.0, 3.7]
	**Shigella**	0.0 [0.0, 3.0]	0.0 [0.0, 3.3]	5.6 [0.0, 17.3]	7.7 [0.0, 24.5]	3.1 [0.0, 7.6]
	**Cholera**	0.0 [0.0, 3.0]	0.0 [0.0, 3.3]	0 [0.0,3.1]	7.7 [0.0, 24.5]	1.6 [0.0, 4.8]
	**Any Bacteria**	**15.8 [0, 33.8]**	**15.3 [0, 38.1]**	**22.2 [0, 43.5]**	**23.1 [0.0, 49.6]**	**19.0 [8.9, 29.1]**
**Protozoa**	**Entamoeba histolytica**	0.0 [0.0, 3.0]	0.0 [0.0, 3.3]	0 [0.0,3.1]	0.0 [0.0, 3.3]	0.0 [0.0, 2.7]
	**Cryptosporidium**	0.0 [0.0, 3.0]	0.0 [0.0, 3.3]	0 [0.0,3.1]	0.0 [0.0, 3.3]	0.0 [0.0, 2.7]
	**Giardia**	5.3 [0.0, 16.3]	0.0 [0.0, 3.3]	11.1 [0.0, 27.2]	15.3 [0.0, 38.1]	8.2 [1.1, 15.2]
	**Any Protozoa**	**5.3 [0.0, 16.3]**	**0.0 [0.0, 3.3]**	**11.1 [0.0, 27.2]**	**15.3 [0.0, 38.1]**	**8.2 [1.1, 15.2]**
**Viruses**	**Rotavirus**	31.6 [8.5, 54.6]	7.7 [0.0, 24.5]	33.3 [9.2, 57.5]	7.7 [0.0, 24.5]	23.1 [12.2, 34.0]
	**Sapovirus**	0.0 [0.0, 3.0]	0.0 [0.0, 3.3]	0 [0.0,3.1]	0.0 [0.0, 3.3]	0.0 [0.0, 2.7]
	**Hepatitis E**	0.0 [0.0, 3.0]	0.0 [0.0, 3.3]	0 [0.0,3.1]	0.0 [0.0, 3.3]	0.0 [0.0, 2.7]
	**Adenovirus**	42.1 [17.7, 66.6]	53.8 [33.5, 85.2]	44.4 [19.0, 69.9]	61.5 [30.9, 92.1]	48.7 [36.0, 61.6]
	**Astrovirus**	0.0 [0.0, 3.0]	0.0 [0.0, 3.3]	0 [0.0,3.1]	0.0 [0.0, 3.3]	0.0 [0.0, 2.7]
	**Norovirus**	31.6 [8.5, 54.6]	30.7 [1.7, 59.8]	22.2 [0.0, 43.5]	7.7 [0.0, 24.5]	23.8 [12.9, 34.7]
	**Any Virus**	**68.4 [45.4, 91.4]**	**69.2 [40.2, 98.3]**	**72.2 [49.3, 95.1]**	**69.2 [40.2, 98.3]**	**69.7 [58.0, 81.5]**
**Any Pathogen**		**73.7 [51.9, 95.5]**	**76.9 [50.4, 100]**	**77.8 [56.5, 99.1]**	**92.3 [75.6, 100]**	**79.2 [68.8, 89.7]**

Rates of enteric pathogens present in stool samples, broken down by season, study arm, and diarrhoea status:*%{95%CI]*.

### 4.5 Estimates of diagnostic accuracy

**Fig. 3:**
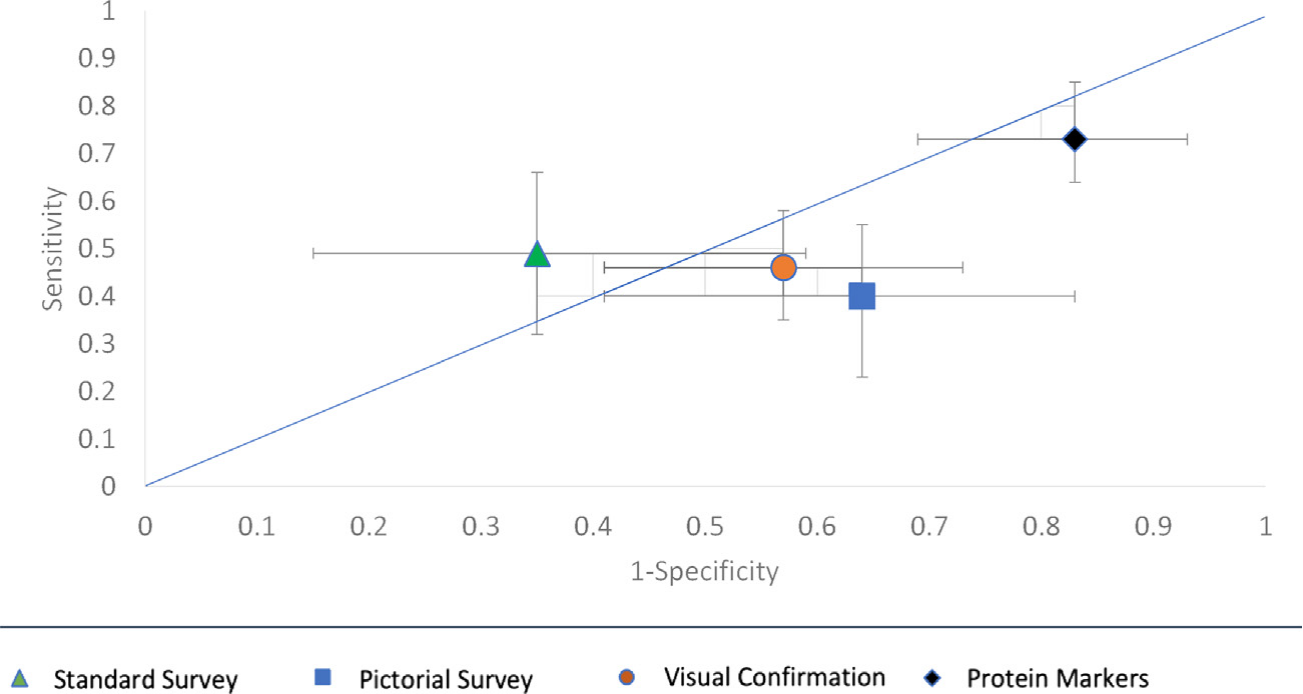
ROC Space plot of Diarrhoea measurement methods as markers of enteric pathogen presence in stool.

Table 3 and Fig. 3 present the sensitivities and specificities of each measure of diarrhoea as a proxy marker of enteric pathogen presence in stool. When looking at the presence of at least one enteric pathogen, proteins had the highest sensitivity, 73% [61,82]. This compares to 49% [32,66] for the standard survey, 40% [25,57] for the pictorial survey, and 46% [34,57] for visual confirmation. We observed little differences in sensitivities when broken down by pathogen type.

**Table 3:** Performance of Diarrhoea measurement methods as indicators of enteric pathogen presence in Stool.

		**Standard Survey**	**Pictorial Survey**	**Visual Confirmation**	**Proteins**
**Any Pathogen**	**Sensitivity**	49% [32, 66]	40% [25, 57]	46% [34, 57]	73% [61, 82]
	**Specificity**	65% [41, 85]	36% [17, 59]	43% [27, 59]	18% [7, 31]
**Bacteria**	**Sensitivity**	48% [23, 72]	42% [20, 66]	56% [38, 73]	84% [68, 94]
	**Specificity**	56% [41, 73]	49% [33, 65]	53% [42, 64]	28% [18, 38]
**Virus**	**Sensitivity**	45% [27, 64]	44% [27, 62]	51% [38, 63]	72% [60, 83]
	**Specificity**	56% [35, 76]	38% [19, 57]	51% [37, 65]	18% [8, 30]
**Protozoa**	**Sensitivity**	50% [1, 99]	33% [4, 78]	50% [16, 84]	75% [35, 97]
	**Specificity**	56% [41, 69]	46% [32, 59]	50% [40, 60]	23% [15, 32]

Diagnostic Performance Indicators (with 95%CIs) of each diarrhoea measure as a proxy marker of Enteric Pathogen Presence in Stool.

Regarding specificity, proteins had the lowest: 18% [7,31]. This compares to 65% [41,85] for the standard survey, 36% [17, 59] for the pictorial survey, and 43% [27,59] for visual confirmation. Again, we find little difference in specificities when breaking down by pathogen type. Overall, we also observe a trade-off between sensitivity and specificity.

Fig. 3 Caption: An ROC Space Plot showing the sensitivities and specificities of all four diarrhoea measurement methods as proxy markers of at least one enteric pathogen being present in stool, with 95%CI error bars. The 45° line represents the point where the method is no better than tossing a coin to identify presence of an enteric pathogen.

### 4.6 Agreement between diarrhoea measures

No pairwise combination of the four proxy markers showed more than a slight level of agreement (0–0.20) above that expected by chance alone (Table 4) [24].

**Table 4:** Pairwise agreements between the four proxy markers.

**Pairwise Combination**	**Kappa (p value)**	**Observed Agreement (%)**	**Expected Agreement (%)**
Visual v Standard Survey	0.14 (0.14)	55.8	48.7
Protein v Standard Survey	0.02 (0.43)	48.2	47.3
Visual v Pictorial Survey	−0.12 (0.83)	44.3	50.2
Protein v Pictorial Survey	−0.05 (0.67)	46.8	69.1
Visual v Protein	−0.10 (0.89)	45.2	50.2

## 5 Discussion

We provide evidence that carer-reported diarrhoea in under-fives from community surveys highly misclassifies enteric pathogen presence in stool. We further found that there was little to no agreement between our four methods of diarrhoea measurement, supporting findings in our recent systematic review [11].

There are multiple sources of misclassification. Firstly, non-pathogenic diarrhoea may result in decreased specificity. There are multiple reasons why a child without an enteric infection may have diarrhoea, including nutritional intolerances [25], antibiotic treatment [26], systemic infections [27], and chronic bowel diseases [28]. While we do not think that these factors explain more than a small proportion of the lack of agreement observed here, there might be more subtle factors. For example, changes in the microbiome due to malnutrition or previous infections may be associated with diarrhoea – an issue to which we return.

Secondly, asymptomatic carriage of enteric pathogens may result in decreased sensitivity. Our results support past work that has found widespread asymptomatic carriage of enteric pathogens, including carriage and excretion after the symptomatic period [29], [30], [31]. Okitsu et al. (2020) found that 80% of healthy children in rural Bangladesh had some enteric pathogen in their stool [30,31]. Carriage is epidemiologically important due to ‘neighbourhood effects.’ A reduction in the total neighbourhood burden of carriage is important in reducing serious morbidity and mortality, and is thus an aim of WASH interventions [32].

Finally, inconsistent reporting may decrease both sensitivity and specificity. Respondents may be inconsistent in their perception of stool, as well as how respondents may respond to the survey. While hard stool and explosive diarrhoea can be easily characterized, young children's stool is often of intermediate consistency and may be classified differently by different observers or by the same observer on different days. We found evidence of the former in the data. We also found that estimates of diarrhoea rates are highly sensitive the way questions are framed in our recent systematic review [11].

Our findings have important implications on the findings of scientific studies. As discussed, if survey based diarrhoea is a poor reflection of the presence of pathogens in stool, diarrhoea is not a good outcome in WASH trials and could contribute to their many null results [3,33]. Of note, two of the three recent large scale WASH trials which used carer reported diarrhoea with a seven-day recall as their endpoint reported null results [6,9,34]. If these trials had used directly measured enteric pathogen presence in stools this may not have been the case. Both Greenland (1996) and Hutcheon and Hanley (2010) demonstrate that misclassification error, particularly that which results in poor specificity, biases estimated intervention effects towards the null [33,35]. We apply Greenland's analysis to Luby *et al*’s (2018) trial in rural Bangladesh [6]. The authors concluded that the intervention did not impact carer-reported diarrhoea rates (adjusted prevalence difference=0.7% [95%CI:−2.4, 3.7]). However, given the sensitivities and specificities we observed, this observation is compatible with a fifty percent reduction in enteric pathogen presence rates (Appendix 8). There are similar findings for other health conditions, including pneumonia and acute respiratory distress syndrome, and it is likely the results also translate to measurements of linear growth [36,37].

Our study has several limitations. First, despite our extensive panel, we may have omitted some important enteric pathogens (perhaps some yet to be discovered), or not detected some pathogens due to the timing of stool collection or the pathogen load being below that able to be detected up by PCR. If survey diarrhoea was a more sensitive predictor of these omitted organisms than of those included, then this would improve the low sensitivities and specificities we observed, although this seems unlikely. Second, we did not quantify the amount of pathogen present. It may be that part of the benefit of WASH interventions lies in reducing the load of pathogens as well as the proportion of children who have pathogens in their stool. If this is thought to be the case it would be important to identify and quantify pathogen load in specifying an optimal outcome variable for WASH trials. Third, we did not analyse the prevalence of co-infection which might also be reduced by WASH interventions. WASH interventions have recently been seen to reduce the rate of co-infection. Fourth, the number of stool analyses is small, though our results suggest low test accuracy within the 95% confidence interval. Fifth, our study was done in one centre only and hence should be replicated. Results may be different in areas with lower given that diarrhoea and enteric infection rates than Cox's Bazar (though we find it hard to see why the results should not apply in similar settings). Sixth, we did not examine other recall periods (including the 7-day recall period used in several trials). Against these limitations our study has strengths in terms of multiple measures of diarrhoea rates and use of ‘reference standard’ PCR testing following internationally recognized quality standards.

Our findings have important practical implications. However, it is important to stress that our findings apply to active surveillance and research practice, not to cases that present clinically in health facilities. Children with bloody or profuse diarrhoea accompanied by dehydration are more likely to have an infection with pathogenic organisms.

In terms of surveillance and research practice, our findings suggest that survey-based diarrhoea is a poor outcome for WASH evaluations. We propose stool microbiology as a better primary outcome and one that is increasingly feasible to measure. This reverses the commonplace logic of making the clinical outcome the primary outcome and treating laboratory outcomes as secondary outcomes or mediators. In this case there are three factors together justify use of stool microbiology as the optimal outcome to measure to evaluate population improvements in WASH. First, diarrhoea is very poorly correlated with microbiology, as has also been seen in another recent paper. Second, the rationale for WASH interventions is to create a barrier to prevent micro-organisms from faeces with pathogens entering the alimentary tract. Lastly, it is not clear that survey-based diarrhoea is an outcome of large personal salience or disutility to parents and children. This contrasts with more severe cases that present clinically, and which are more obviously important to patients and families.

One could argue for a measurement of “clinically significant” or “severe” diarrhoea as the optimal outcome for evaluating water and sanitation improvement. These cases are much more likely than survey-based cases to represent active infection and they are of undisputed importance to child health and development. This measurement does have drawbacks. First, if defined by presentation to a clinic or hospital it is a rare outcome as referenced in the introduction and may therefore be imprecise unless sample sizes are large. Second, it may be open to selection bias if intervention recipients are more or less likely than controls to seek treatment. Third, it will omit asymptomatic carriers who are a crucial reservoir of pathogenic bacteria. That said, the presence of infectious organisms in the gut is a mediator of clinical illness, so a combined approach of stool micro-biology and assessment of cases that present clinically would seem a scientifically sound approach. However, it is worth noting that there are many different types of microbiological assays for stool micro-biology, including rapid diagnostic tests; as well as sampling approaches, such as pooling stool samples in lower prevalence areas and examination of sludge [38]. Given that microbiological testing is more costly than clinical observation, more research will be needed to optimise value for money when stool microbiology is more extensively used in WASH trials.

Our research leaves an important question open. For all that survey based carer reported diarrhoea is not an accurate test for the pathogens that WASH may prevent, it is premature to conclude that survey diarrhoea rates tell us nothing at all. Diarrhoea is widely reported, even by our third party ‘experts’ who examined the samples. Additionally, inflammatory marker levels were raised in most samples. It is increasingly clear that the more subtle changes in the microbiome are important, including those that can worsened by malnutrition and improved by pro-biotic nutrition [39]. It seems likely that enteric health is more than just the absence of pathogens. It might be the case that interventions that nurture a healthy microbiome over the weaning period could leave an indelible effect of subsequent intestinal health, a possibility that the authors are actively exploring in a MRC funded trial in Mali [40].

## Contributors

- Rego, Watson, and Islam were involved in study conception, design, field data collection, lab analysis, statistical analysis, and writing, and all had access to and handled the raw data.
- Yunus, Gill, Faruque, Khan, and Lilford were involved in study conception, design, field data collection, lab analysis, statistical analysis, and writing, but did not have access to or handled raw data
- Ul-Alam was involved in field data collection, lab analysis, and writing, and all had access to and handled the raw data.
- Alam, Chowdhury, and Haider were involved in field data collection, lab analysis, and writing, but did not have access to or handled raw data
- Abdullah was involved in field data collection and writing
- Hofer was involved in writing and interpretation of data. All authors together decided to submit for publication.

## Data sharing statement

Data can be accessed immediately on reasonable request through emailing the corresponding author. Rego, Watson, Islam and Ul-Alam had access to and handled the raw study data.

## Funding

The project was funded by the National Institutes for Health Research Global Health Research Unit on Improving Health in Slums (16/136/87) and by the University of Warwick.

## Declaration of Competing Interest

Travel for the project, as well as salaries for RJL, SW, and RR were funded by the National Institutes for Health Research Global Health Research Unit on Improving Health in Slums (16/136/87) using UK aid from the UK Government. The views expressed in this publication are those of the author(s) and not necessarily those of the NIHR or the Department of Health and Social Care. The funder had no role in study design, implementation, or interpretation. RJL is also director of the NIHR Applied Research Centre and is supported by NIHR ARC West Midlands.

## Appendix Supplementary materials

Image, application 1

Image, application 2

Image, application 3

Image, application 4

Image, application 5

Image, application 6

Image, application 7

Image, application 8
