# Supplementary material for: A comparison of traditional diarrhoea measurement methods with microbiological and biochemical indicators: A cross-sectional observational study in the Cox's Bazar displaced persons camp
Source: eClinicalMedicine. 2021 Nov 20;42:101205. doi: 10.1016/j.eclinm.2021.101205 (PMC8608865; doi:10.1016/j.eclinm.2021.101205)
Supplement: Supplementary file 1 [file mmc1.docx]

# Appendix 1: Randomisation

Selection of households and the randomisation between arms was conducted by RR the day before data collection. We used a variant of the extended program on immunization (EPI) sampling method, in which a series of random angles and distances from a predetermined central point were generated, selecting households closest to the random point^41^. Prior to the start of the day, a list of houses was generated using the EPI sampling method, with an arm randomly assigned using simple random sampling via the RAND function in Microsoft Excel. Selection of households from whom to collect stool was also conducted through simple random sampling using the RAND function in Microsoft Excel, taking into account the expected number of households reporting diarrhoea (10%) and not reporting diarrhoea (90%). Given the nature of the study, there was no blinding or allocation concealment.
