# Supplementary material for: A comparison of traditional diarrhoea measurement methods with microbiological and biochemical indicators: A cross-sectional observational study in the Cox's Bazar displaced persons camp
Source: eClinicalMedicine. 2021 Nov 20;42:101205. doi: 10.1016/j.eclinm.2021.101205 (PMC8608865; doi:10.1016/j.eclinm.2021.101205)
Supplement: Supplementary file 2 [file mmc2.docx]

# Appendix 2: Surveys

Data Collection Log Book (Separated Data)

| Name |  |
| --- | --- |
| Phone Number |  |
| Household Barcode | SCAN BARCODE |

Survey Data (Separate from Log Book)

|  | | | | | | | | | | | | | | | | | | | | | | |  |  |  |
| --- | --- | --- | --- | --- | --- | --- | --- | --- | --- | --- | --- | --- | --- | --- | --- | --- | --- | --- | --- | --- | --- | --- | --- | --- | --- |
| RA | 1 | | 2 | | | 3 | | | | | 4 | | |  | | |  | | |  | |  |  |  |  |
| EA |  | |  | | |  | | | | |  | | |  | | |  | | |  | |  |  |  |  |
| Date |  | |  | | |  | | | | |  | | |  | | |  | | |  | |  |  |  |  |
| Household Barcode | SCAN BARCODE | |  | | |  | | | | |  | | |  | | |  | | |  | |  |  |  |  |
| **Resident Information (all respondents)** | | | | | | | | | | |  | | |  | | |  | | |  | |  |  |  |  |
| Name | Age | | Sex | | | Education Level | | | | | Occupation | | | Health Conditions | | | Time Spent in Bangladesh | | | Relationship to Respondent | |  |  |  |  |
| RESPONDANT |  | |  | | |  | | | | |  | | |  | | |  | | |  | |  |  |  |  |
| SPOUSE IF PRESENT |  | |  | | |  | | | | |  | | |  | | |  | | |  | |  |  |  |  |
| Child/Other Family |  | |  | | |  | | | | |  | | |  | | |  | | |  | |  |  |  |  |
| Child/Other Family |  | |  | | |  | | | | |  | | |  | | |  | | |  | |  |  |  |  |
| Child/Other Family |  | |  | | |  | | | | |  | | |  | | |  | | |  | |  |  |  |  |
| Child/Other Family |  | |  | | |  | | | | |  | | |  | | |  | | |  | |  |  |  |  |
| Child/Other Family |  | |  | | |  | | | | |  | | |  | | |  | | |  | |  |  |  |  |
| Child/Other Family |  | |  | | |  | | | | |  | | |  | | |  | | |  | |  |  |  |  |
| **Household Information (all respondents)** | | | | | | | | | | | | | |  | | |  | | |  | |  |  |  |  |
| What is the main source of drinking water for your household? | Piped water into home | | Piped water into plot | | | Piped water to tap | | | | | Borehole | | | Dug well | | | Spring | | | Rain | | Truck | Cart | Surface Water | Bottled Water |
| Where is that water source located | In my home | | In my plot | | | In a public space | | | | |  | | |  | | |  | | |  | |  |  |  |  |
| How long does it take you to get there? |  | | Minutes | | |  | | | | |  | | |  | | |  | | |  | |  |  |  |  |
| Do you share this with other households? | Yes | | No | | |  | | | | |  | | |  | | |  | | |  | |  |  |  |  |
|  | If yes, how many | |  | | | Households | | | | |  | | |  | | |  | | |  | |  |  |  |  |
|  | If yes, how long do you have to wait for water | |  | | | Minutes | | | | |  | | |  | | |  | | |  | |  |  |  |  |
| Do you do anything to make this water safe to drink? | Yes | | No | | |  | | | | |  | | |  | | |  | | |  | |  |  |  |  |
|  | If yes, what? | | Boil | | | Bleach/Chlorine | | | | | Cloth Strain | | | Filter | | | Solar disinfection | | | Sit and Settle | | Other: |  |  |  |
| Can I see where you use the toilet? | Yes | | No | | |  | | | | |  | | |  | | |  | | |  | |  |  |  |  |
|  | If yes, describe: | | Flush toilet to sewer | | | Pit Latrine | | | | | VIP Latrine | | | Flush toilet to Latrine | | | Composting Toilet | | | Bucket Toilet | | Open Defecation | Other: |  |  |
| How many people use this toilet |  | | People | | |  | | | | |  | | |  | | |  | | |  | |  |  |  |  |
| Is this toilet separated by sex | Yes | | No | | |  | | | | |  | | |  | | |  | | |  | |  |  |  |  |
| Do you feel safe using this toilet during the day? | Yes | | No | | |  | | | | |  | | |  | | |  | | |  | |  |  |  |  |
| Do you feel safe using this toilet when dark? | Yes | | No | | |  | | | | |  | | |  | | |  | | |  | |  |  |  |  |
| How do you wash your hands? | Water alone | | Water with Soap | | | Alcohol Based Sanitizer | | | | | Non-Alcohol Based Sanitizer | | | Does not wash hands | | | Other: | | |  | |  |  |  |  |
| When do you wash your hands? (all that apply) | After Defecation | | After cleaning a child’s stool | | | Before Feeding a Child | | | | | Before Eating | | | Before preparing food | | | Other: | | |  | |  |  |  |  |
| Do you own livestock? | Yes | | | | | | No | | | | | | |  | | |  | | |  | |  |  |  |  |
| Where do you normally seek healthcare? |  | |  | | |  | | | | |  | | |  | | |  | | |  | |  |  |  |  |
| OBSERVE MATERIAL OF FLOOR | Natural Floor | | Wood | | | Bamboo | | | | | Tarp | | | Finished Floor | | | Other: | | |  | |  |  |  |  |
| OBSERVE MATERIAL OF ROOF | No roof | | Thatch | | | Bamboo | | | | | Wood | | | Cardboard | | | Tin | | | Ceramic | | Cement | Tent | Other: |  |
| OBSERVE WATER STORAGE VESSEL | Open Bucket | | Closed bucket | | | Bottle | | | | | Jerry Can | | | Bucket | | | Other: | | |  | |  |  |  |  |
|  |  | |  | | |  | | | | |  | | |  | | |  | | |  | |  |  |  |  |
| **Basic Survey (50% of respondants)** | | | | | | | | | | |  | | |  | | |  | | |  | |  |  |  |  |
| *IDENTIFY OLDEST CHILD UNDER 5* |  | |  | | |  | | | | |  | | |  | | |  | | |  | |  |  |  |  |
| Is this child breastfed? | Yes – Exclusively | | Yes – partially | | | No | | | | |  | | |  | | |  | | |  | |  |  |  |  |
| Has this child had 3 or more loose or watery stools any day in the past 2 weeks? | Yes | | No | | |  | | | | |  | | |  | | |  | | |  | |  |  |  |  |
| Has this child had blood in their stool in the past 2 weeks? | Yes | | No | | |  | | | | |  | | |  | | |  | | |  | |  |  |  |  |
|  | If yes to either, did you seek treatment for either of these? | | Yes | | | No | | | | |  | | |  | | |  | | |  | |  |  |  |  |
|  |  | | If yes, where? | | |  | | | | |  | | |  | | |  | | |  | |  |  |  |  |
|  |  | |  | | |  | | | | |  | | |  | | |  | | |  | |  |  |  |  |
| **Enhanced Survey (50% of respondants)** | | | | | | | | | | |  | | |  | | |  | | |  | |  |  |  |  |
| *IDENTIFY OLDEST CHILD UNDER 5* |  | |  | | |  | | | | |  | | |  | | |  | | |  | |  |  |  |  |
| Is this child breastfed? | Yes – Exclusively | | Yes – partially | | | No | | | | |  | | |  | | |  | | |  | |  |  |  |  |
| Mid-Upper Arm Circumference |  | | cm | | |  | | | | |  | | |  | | |  | | |  | |  |  |  |  |
| *SHOW AMSTERDAM STOOL CHART*Has the child had any of these stools in the past 2 weeks | Consistency | A | | B | C | | | D |  | | |  | | |  | | |  | | | | | | | |
|  | Colour | 1 | | 2 | 3 | | | 4 | 5 | 6 | | |  | | |  | | |  | |  | | | | |
| How long ago? | 1 | | 2 | | | 3 | | | | | 4 | | | 5 | | | 6 | | | 7 | | Other: |  |  |  |
| How many times on the worst day? | 1 | | 2 | | | 3 | | | | | 4 | | | 5 | | | 6 | | | 7 | | Other: |  |  |  |
| How many days did it last? | 1 | | 2 | | | 3 | | | | | 4 | | | 5 | | | 6 | | | 7 | | Other: |  |  |  |
| Did this child have fever in the past 2 weeks? | Yes | | No | | |  | | | | |  | | |  | | |  | | |  | |  |  |  |  |
| Has this child had blood in their stool in the past 2 weeks? | Yes | | No | | |  | | | | |  | | |  | | |  | | |  | |  |  |  |  |
| Did this child have vomiting in the past 2 weeks | Yes | | No | | |  | | | | |  | | |  | | |  | | |  | |  |  |  |  |
| Has this child been eating properly in the past 2 weeks | Yes | | No | | |  | | | | |  | | |  | | |  | | |  | |  |  |  |  |
|  | If yes to either, did you seek treatment for either of these? | | Yes | | | No | | | | |  | | |  | | |  | | |  | |  |  |  |  |
|  |  | | If yes, where? | | |  | | | | |  | | |  | | |  | | |  | |  |  |  |  |
|  | If yes to either to these, did you provide treatment? | | Yes | | | No | | | | |  | | |  | | |  | | |  | |  |  |  |  |
|  |  | | If yes, what? | | | Commercial ORS | | | | | Homemade ORS | | | Zinc | | | Traditional medicine | | | Antibiotics | | Other: |  |  |  |
| Has this child had any unexplained rashes or redness in the past 2 weeks? | Yes | | No | | |  | | | | |  | | |  | | |  | | |  | |  |  |  |  |
| Has this child had any unexplained pink eye or discharge from eyes in the past 2 weeks? | Yes | | No | | |  | | | | |  | | |  | | |  | | |  | |  |  |  |  |
|  | If yes to either, did you seek treatment for either of these? | | Yes | | | No | | | | |  | | |  | | |  | | |  | |  |  |  |  |
|  |  | | If yes, where? | | |  | | | | |  | | |  | | |  | | |  | |  |  |  |  |
|  |  | |  | | |  | | | | |  | | |  | | |  | | |  | |  |  |  |  |
