# Supplementary material for: A comparison of traditional diarrhoea measurement methods with microbiological and biochemical indicators: A cross-sectional observational study in the Cox's Bazar displaced persons camp
Source: eClinicalMedicine. 2021 Nov 20;42:101205. doi: 10.1016/j.eclinm.2021.101205 (PMC8608865; doi:10.1016/j.eclinm.2021.101205)
Supplement: Supplementary file 3 [file mmc3.docx]

# Appendix 3: Amsterdam Stool Chart


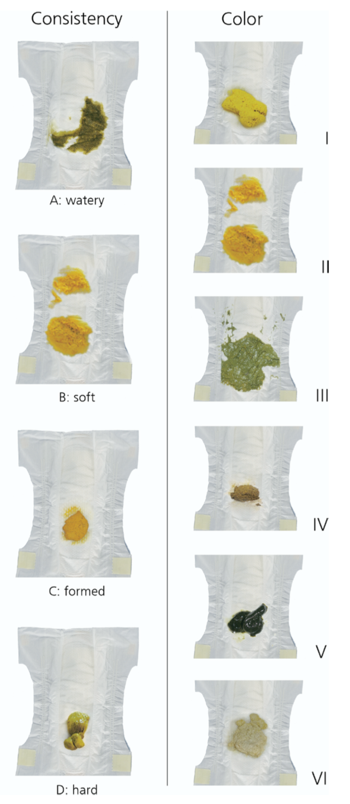


Amsterdam Stool Chart, with showing stools in diapers with Consistency A (Watery ) to D (Hard), and colour I (yellow) to VI (pale gray) (From Bekkali et al, 2009)
