# Supplementary material for: A comparison of traditional diarrhoea measurement methods with microbiological and biochemical indicators: A cross-sectional observational study in the Cox's Bazar displaced persons camp
Source: eClinicalMedicine. 2021 Nov 20;42:101205. doi: 10.1016/j.eclinm.2021.101205 (PMC8608865; doi:10.1016/j.eclinm.2021.101205)
Supplement: Supplementary file 4 [file mmc4.docx]

# Appendix 4: Lab Methods

## Bacteriology Method

The extracted DNA was subjected for PCR amplification for the presence of various bacterial pathogenic genes. We used internal (positive and negative) controls for PCR and nucleic acid exctraction using the InviMeg kit to maintain quality. The genes included are attaching and effacing gene (*eae*), bundle forming pilus (*bfp*), heat labile (lt), heat stable (st) by multiplex PCR assay. The template DNA (3 µl) was suspended in 22 µl of reaction mix containing 12.5 µl 2X PCR Mastermix, 0.44 µl primers for each of *eae, bfp, est, elt* genes. PCR cycling conditions consisted of initial denaturation at 96^o^C for 4 min, followed by 35 cycles (each cycle consists of denaturation at 95^o^C for 20s, annealing at 57^o^C for 20s and extension at 72^o^C for 1 min) and 5min of final extension at 72^o^C. Multiplex PCR was carried out for Shiga toxin (stx1 & stx2) according to the procedure described earlier^[[1]](#footnote-1)^ and for the invasion plasmid antigen H gene (*ipaH*) was performed according to published procedures^[[2]](#footnote-2)^. Multiplex PCR was carried out with initial denaturation at 94°C for 5 min, followed by 35 cycles (each cycle consists of denaturation at 94^o^C for 1min, annealing at 58°C for 1min, and extension at 72°C for 2min) and final extension at 72°C for 10min for the detection of Cholera toxin (*ctx*) and Cholera toxin transcriptional activator (*toxR*)^[[3]](#footnote-3)^. After completing the Multiplex PCR, all the suspected positive samples were then subjected to simplex PCR for confirmation with the corresponding primers. To detect *Campylobacter* genus, 16s rRNA gene was detected by simplex PCR using specific primers where the initial denaturation was at 95^o^C for 10 min, followed by 35 cycles (each consists of denaturation at 95^o^C for 30s, annealing at 59^o^C for 1min and extension at 72^o^C for 1min) and then final extension at 72^o^C for 10 mins^[[4]](#footnote-4)^.

15 min at 95C, a cycle of

three steps repeated 30 times [45 s at 94C, 1 min at

annealing temperature (Table 2) and 1 min at 72C], and

ﬁnally, 10 min at 72C.

15 min at 95C, a cycle of

three steps repeated 30 times [45 s at 94C, 1 min at

annealing temperature (Table 2) and 1 min at 72C], and

ﬁnally, 10 min at 72C.

### Primer Sequences

| **Sl. No.** | **Reference paper & No.** | **Species** | **Target gene** | **Primer sequence (5′–3′)** | **Annealing temp. (^o^C)** | **Amplification (bp)** |
| --- | --- | --- | --- | --- | --- | --- |
| 1 | Talukdar et al., 2013 & (1) | Enteropathogenic *E*. *coli* | *eae* | eae-F  CCCGAATTCGGCACAAGCATAAGC  eae-R CCCGGATCCGTCTCGCCAGTATTCG | 57 | 881 |
| 2 | Talukdar et al., 2013 & (1) | Enteropathogenic *E*. *coli* | *bfpA* | bfpA-F  GGAAGTCAAATTCATGGGGG  bfpA-R  GGAATCAGACGCAGACTGGT | 57 | 300 |
| 3 | Talukdar et al., 2013 & (1) | Enterotoxigenic  *E*. *coli* | *estA* | estA-F  GCTAAACCAGTAGAGGTCTTCAAAA  estA-R  CCCGGTACAGAGCAGGATTACAACA | 57 | 147 |
| 4 | Talukdar et al., 2013 & (1) | Enterotoxigenic  *E*. *coli* | *eltB* | eltB-F  CACACGGAGCTCCTCAGT C  eltB-R  CCCCCAGCCTAGCTTAGTTT | 57 | 508 |
| 6 | Talukdar et al., 2013 & (1) | Shiga toxin producing *E*. *Coli* | *stx1* | stx1-F CACAATCAGGCGTCGCCAGCGCACTTGCT  stx1-R TGTTGCAGGGATCAGTGGTACGGGGATGC | 58 | 606 |
| 7 | Talukdar et al., 2013 & (1) | Shiga toxin producing *E*. *Coli* | *stx2* | stx2-F CCACATCGGTGTCTGTTATTAACCACACC  stx2-R GCAGAACTGCTCTGGATGCATCTCTGGTC | 58 | 372 |
| 8 | Rivera et al., 2001  & (7) | *V. cholerae* | *ctxA* | ctxA-F  CGGGCAGATTCTAGACCTCCTG  ctxA-R  CGATGATCTTGGAGCATTCCCAC | 58 | 564 |
| 9 | Rivera  et al., 2001 & (7) | *V. cholerae* | *toxR* | toxR-F  CCTTCGATCCCCTAAGCAATAC  toxR-R  AGGGTTAGCAACGATGCGTAAG | 58 | 779 |
| 10 | Denis et al., 1999 & (8) | *Campylobacter spp.* | 16s rRNA | MD16S2-F  ATCTAATGGCTTAACCATTAAAC  MD16S2-R  GGACGGTAACTAGTTTAGTATT | 59 | 857 |
| 11 | Paton et al., 1998 & (6) | *Shigella spp.* | *ipaH* | ipa-F  CTCGGCACGTTTTAATAGTCTGG  ipa-R  GTGGAGAGCTGAAGTTTCTCTGC | 58 | 933 |
| 12 | Shanmugasamy et al., 2011 & (9) | *Salmonella spp* | *invA* | inV-F  GTGAAATTATCGCCACGTTCGGGCAA  inv-R  TCATCGCACCGTCAAAGGAACC | 64 | 284 |

## Viral Identification

For the amplification of viral pathogens, total nucleic acid had first extracted from the fecal samples using magnetic particle based InviMag^®^ Pathogen Kit (STRATEC Molecular GmbH, Germany) according to the manufacturer’s instructions, with internal positive and negative controls. All procedures were done inside the BSC II. Switched on the BSC II and run for 10 minutes before starting the work. Before starting NA extraction the work surface in the BSC II was cleaned with 0.5% Bleach and wiped with RNaseZap/ 0.1M HCl solution to remove any traces of RNase enzyme.

Labeled 1.5 or 2 ml microcentrifuge tube for each primer/probe set within the PCR work station. The preparation of master mix for each assay as follows:

### Master Mix

| **Components** | **AgPath** | **Bio-Rad** |
| --- | --- | --- |
| 2× RT-PCR Buffer | N x 12.5 μl | N x 10 μl |
| 25× Enzyme Mix | N x 1 μl | N x 0.4μl |
| Detection Enhancer | N x 1 μl | N x 0 μl |
| Forward primer (40 μM) | N x 0.5 μl | N x 0.5 μl |
| Reverse primer (40 μM) | N x 0.5 μl | N x 0.5 μl |
| Probe (10 μM) | N x 0.5 μl | N x 0.5 μl |
| Nuclease-free water | N x 4 μl | N x 4 μl |
| **Total volume** | **N x 20 μl** | **N x 16 μl** |

**N= Number of Reaction*

Thermal cycling protocol in the qRT-PCR machine for detection of stool pathogens are as follows:

• 10 min at 45°C

• 3 min at 95°C

• 45 cycles of 15 sec. at 95°C and 1 min at 55°C (Data Acquisition)

Both the linear and logarithmic curves for actual PCR amplification are reviewed. We have set the threshold at the mid-point of the logarithmic position of the curve specific for each target. We have reviewed the linear and logarithmic curves. Then noted the lowest Ct value. Our analysis of the each target is done by reviewing the each target curves. Then in the interpretation. NTCs (Negative Template Controls) and Mock (if included) should not show fluorescent curves above the threshold line (Ct). If one of the NTCs crosses the threshold, the entire experiment is invalid and should be repeated. VTC (Viral Template Controls) reactions should exhibit fluorescence curves that cross the threshold line (Ct ranging from 24-28).

### Primer Sequences

| **Gene** |  | **Sequence (5’→3’)** |
| --- | --- | --- |
| **Rota NSP3** | Rota F | CAG TGG TTG ATG CTC AAG ATG GA |
|  | Rota R | TCA TTG TAA TCA TAT TGA ATA CCC A |
|  | Rota P | FAM-ACA ACT GCA GCT TCA AAA GAA GWG T-BHQ1 |
| **Noro**  **GI** | Cog 1F | CGYTGGATGCGITTYCATGA |
|  | Cog 1R | CTTAGACGCCATCATCATTYAC |
|  | Ring 1 | FAM – TGGACAGGRGAYCGC – MGB |
| **Noro**  **GII** | Cog 2F | CARGARBCNATGTTYAGRTGGATGAG |
|  | Cog 2R | TCGACGCCATCTTCATTCACA |
|  | Ring 2 | Cy5 – TGGGAGGGCGATCGCAATCT – BHQ2 |
| **HepA 5’UTR** | HepA F | TCACCGCCGTTTGCCTAG |
|  | HepA R | GGAGAGCCCTGGAAGAAAG |
|  | HepA P | FAM-CCTGAACCTGCAGGAATTAA-MGB |
| **HepE ORF2** | HepE F | GGTGGTTTCTGGGGTGAC |
|  | HepE R | AGGGGTTGGTTGGATGAA |
|  | HepE P | FAM-TGATTCTCAGCCCTTCGC-MGB |
| **Sapo ORF1** | SaV124F | GAY CAS GCT CTC GCY ACC TAC |
|  | SaV1F | TTG GCC CTC GCC ACC TAC |
|  | SaV5F | TTT GAA CAA GCT GTG GCA TGC TAC |
|  | SaV1245R | CCC TCC ATY TCA AAC ACT A |
|  | SaV124TP | FAM-CCR CCT ATR AAC CA-MGB-NQF |
|  | SaV5TP | FAM–TGC CAC CAA TGT ACC A-MGB-NQF |
| **AsV 5’UTR ORF1** | AsV F | TCTYATAGACCGYATTATTGG |
|  | AsV R | TCAAATTCTACATCATCACCAA |
|  | AsV P | Cy5-CCCCADCCATCATCATCTTCATCA-BHQ3 |
| **Adeno hexon gene** | Ad_F | GCC CCA GTG GTC TTA CAT GCA CAT C |
|  | Ad_R | GCC ACG GTG GGG TTT CTA AAC TT |
|  | Ad_P | FAM-TGCACCAGACCCGGGCTCAGGTACTCCGA-TAMRA |

## Protozoa Panel Assay

Necessary Reagents:

- Bio-Rad iQ Multiplex Powermix, store at -20°C (Cat. No. 172-5848 or 172-5849)
- Protozoa Panel Primer/Probe Mix
  (Prepared and Quality Control checked by UVA team)
- Nuclease-Free Water
- Access to a CFX96
- Calibrated pipettes p10; p200, p10 and p200 multichannel pipettes
- Disposable Reagent Reservoirs (sterile DNase and RNase free) (Cat 140248-A1)
- Hard-Shell® Low-Profile Thin-Wall 96-Well Skirted PCR Plates (Bio-Rad Cat HSP 9635)
- Microseal B Optically clear Adhesive tape (Bio-Rad Cat HSP MSB-1001)
- HandyFuge™ Plate Centrifuge (or similar) (Fisher Scientific Cat NC0387427)

**Important Notes**

Nuclease free water to be prepared in 1ml aliquots and Primer/Probe Mix to be aliquoted in amounts sufficient for one days worth of reactions to minimize contamination problems.

Reactions to be set up in a pre-PCR clean area

**Day of experiment**

Daily Cleaning of Pre‐PCR Area

A daily cleaning of the pre‐PCR area using a 0.5% Sodium Hypochlorite (10% Bleach)

solution helps to eliminate PCR product that has entered the pre‐PCR area.

Identify pre‐PCR areas that pose the highest risk of contamination, and clean these areas with a 0.5% Sodium Hypochlorite (10% Bleach) solution before beginning any pre‐PCR processes. High‐risk areas might include, but are not limited to, the following items:

• Benchtops

• Door handles

• Refrigerator/freezer door handles

• Computer mouse

• Keyboards

A no template control plus positive cryptosporidium, Entamoeba histolytica and Giardia samples to be run for every reaction mix prepared and results noted in lab notebook

Reaction Mix Preparation

| **Component** | **Starting conc.** | **Volume (µL)** | **Reaction Mix prepared for**  **______ rxn (μL)** | **Final conc.** |
| --- | --- | --- | --- | --- |
| iQ Powermix | 2x | 12.5 |  | 1x |
| Primer/Probe Mix | Approx. 3.3x | 7.5 |  | 1x |
| Nuclease-Free H_2_O | n/a | 3 |  | n/a |
| Sample | Variable | 2 |  | Variable |
|  | **Total Volume** | **25** |  |  |

Master mix to be decanted into a reagent reservoir, and 24μl dispensed into a PCR plate using multichannel pipette. DNA to either be very carefully added one μl per well or a “master DNA plate” prepared with 10 μl aliquot of ever DNA sample to be tested and two μl transferred to the reaction plate using the p10 multichannel pipettor.

Single qPCR reaction run for each sample initially – all positive samples to be repeated the next day.

Cover wells with optically clear tape – avoid touching the tape surface with ungloved hands as this will transfer oils that will distort optical properties.

Spin plate briefly to make sure all sample is at the bottom of the well

If running this for the first time peruse Overall PCR SOP CFX document

**Protocol for loading sample IDs**

Open the CFX Manager Program

Create a new run

Click on Protocol tab, click on “Open” and load Protozoan.prcl (saved from the collab folder)

Click OK

Check Thermocycling Conditions

|  | Temperature | Time | Purpose |
| --- | --- | --- | --- |
| 1 cycle | 95°C | 3 minutes | Initial denaturation and Taq activation |
| 40 cycles | 95°C | 10 seconds | PCR amplification and target detection |
|  | 60°C | 1 minute* |  |

*Plate read here

Then click on the ‘Plate” tab, then click on “select existing”

Load “QuickPlate_96 wells_four_Channels.pltd” (saved from the collab folder)

Then select “edit selected”

On the next screen click on “spreadsheet view/importer tab”

Click on “import” button and find your run ID.cvs file and load

Next find the “Flors” button and click on the next Fluorophore i.e. Texas Red

Click on “import” button again find your run ID.cvs file and open

Repeat until all 4 Fluorophores loaded

Return to plate tab

Enter target names next to appropriate fluorophore

Targets and Fluorophores

| Fluorophore | Target |
| --- | --- |
| FAM | *E. histolytica* |
| VIC | *Giardia* |
| Texas Red | *Cryptosporidium* |
| Quasar 670 | PhHV |

Save as “run ID”.pltd and click on OK

Load your plate into the CFX96, and then click on Start Run. You will be required to name your run and specify a save location before the protocol is started. Use the run ID name developed using the prescribed naming format.

Reaction Report to be printed at end of each run and entered into lab notebook

A copy of the run file is to be uploaded into the technician’s Dropbox on the UVA collab site “Transmission of Crypto” in order that double data entry of study results can be done at UVA.

Technician analyzes results according to the Baseline-Threshold SOP document and enters results into the study database

Targets and Fluorophores

| Target | Fluorophore |
| --- | --- |
| *Cryptosporidium* | Texas Red |
| *E. histolytica* | FAM |
| *Giardia* | VIC |
| PhHV | Quasar 670 |

Analysis Cut-Off Values

| Target | Ct Cut-off for positivity |
| --- | --- |
| Cryptosporidium | <=36 |
| *E. histolytica* | <=36 |
| Giardia | <=36 |
| PhHV | None |

References

A laboratory-developed TaqMan Array Card for simultaneous detection of 19

enteropathogens.

Jie Liu, Jean Gratz, Caroline Amour, Gibson Kibiki, Stephen Becker, Lalitha Janaki, Jaco J

Verweij, Mami Taniuchi, Shihab U Sobuz, Rashidul Haque, Doris M Haverstick, Eric R Houpt;

Journal of clinical microbiology; 2013

Real-time PCR detection and speciation of Cryptosporidium infection using Scorpion

probes.

Suzanne E Stroup, Shantanu Roy, John Mchele, Venance Maro, Simon Ntabaguzi, Abdullah

Siddique, Gagandeep Kang, Richard L Guerrant, Beth D Kirkpatrick, Ronald Fayer, Joel Herbein,Honourine Ward, Rashidul Haque, Eric R Houpt;Journal of medical microbiology; 2006

## Measurement of Calprotectin in stool samples:

Calprotectin in fecal samples were measure by ELISA procedure. The faces extract samples were diluted at 1:100 ratio, with 10 μl sample added to 990 μl of sample dilution buffer. The dilution mixtures were vortexed to obtain uniform suspension. Samples, standards, and controls (100 μl each) were added in duplicate ELISA wells. The plate containing the wells were covered in sealing foil, placed in a horizontal plate shaker with 500-700 rpm, and incubated at room temperature for 40-45 minutes. After incubation, the liquid from each well was removed and the wells were washed with 300 μl washing solution. The washing solution was again removed and another batch of 300 μl washing solution was added in each well. This process was repeated three times. After the final wash, the remaining liquid was removed thoroughly. The enzyme conjugate vial was mixed gently without shaking. In each well, 100 μl of the conjugate enzyme was added using a multichannel pipette. The plate was then sealed with sealing foil and incubated at room temperature for 45 minutes in a horizontal plate shaker with approximately 500-700 rpm rotation speed. The wells were washed as described previously with washing solution, then 100 μl of enzyme substrate solution was added in each well. The plate was then incubated at room temperature for 20-30 minutes without shaking and protected from light. The optical density of each well was measured using an ELISA reader (BioTek™ Eon™ Microplate Spectrophotometer). The plate was briefly shaken for 2-3 second before reading the optical density. For quality control, new standard carve was created with each run. Additionally, positive control was included in each run.

The reference values for calprotectin in stool samples were adopted from literature (Johne et al., n.d.; Røseth et al., 1992). The normal value of calprotectin in the stool sample of a healthy child was between 5-50 mg/kg, a value over 50 mg/kg was considered a positive value. A value between 200 – 40,000 mg/kg would indicated active inflammatory bowel disease.

### Measurement of Lactoferrin in stool samples:

The measurement of lactoferrin in stool sample followed a similar procedure of ELISA. In an ELISA plate with wells, 100 μl of diluted samples were added. In the same plate, dilution buffer (blank) and standard lactoferrin solutions were added. The plate was then incubated at 37°C for 1 hour without shaking. After incubation, 350 μl of wash solution was added in each well, then the liquid was removed. This washing procedure was followed five time. After the final washing, the plate was inverted and was tapped strongly against a paper towel to ensure the removal of residual liquid. After washing, 100 μl of biotin labeled antibody was added in each well. The plate was then incubated for 1 hour at 37°C without shaking. Following the procedure stated earlier, the wells were washed five times with 350 μl wash solution for each well. The plate was pressed against a paper towel to remove residual liquid after the last washing. Next, 100 μl of Streptavidin-HPR conjugate was added in each well and incubated at 37°C for 30 minutes without shaking. The wells were again washed five times following the same steps as previous washing procedure, including the removal of residual liquids by paper towel. Finally, 100 μl of substrate solution was added to each well. The plate was covered in aluminum foil to prevent exposure to direct light, and the it was incubated at room temperature for 10 minutes. To control the development of color, 100 μl of stop solution was added in each well after incubation. The absorbance of each well was measured using a microplate reader at 450 nm (BioTek™ Eon™ Microplate Spectrophotometer). To obtain accurate readings, the absorbance was measured within 5 minutes after adding stop solution.

1. Talukdar, P. K., Rahman, M., Rahman, M., Nabi, A., Islam, Z., Hoque, M. M., ... & Islam, M. A. (2013). Antimicrobial resistance, virulence factors and genetic diversity of Escherichia coli isolates from household water supply in Dhaka, Bangladesh. *Plos one*, *8*(4), e61090. [↑](#footnote-ref-1)
2. Toxigenic Escherichia coli by using multiplex PCR assays for stx1, stx2, eaeA, enterohemorrhagic E. coli hlyA, rfbO111, and rfbO157. *Journal of clinical microbiology*, *36*(2), 598. [↑](#footnote-ref-2)
3. Rivera, I. N., Chun, J., Huq, A., Sack, R. B., & Colwell, R. R. (2001). Genotypes associated with virulence in environmental isolates of Vibrio cholerae. *Applied and Environmental Microbiology*, *67*(6), 2421. [↑](#footnote-ref-3)
4. Denis, M., Soumet, C., Rivoal, K., Ermel, G., Blivet, D., Salvat, G., & Colin, P. (1999). Development of am‐PCR assay for simultaneous identification of Campylobacter jejuni and C. coli. *Letters in applied microbiology*, *29*(6), 406-410. [↑](#footnote-ref-4)
