# Supplementary material for: A comparison of traditional diarrhoea measurement methods with microbiological and biochemical indicators: A cross-sectional observational study in the Cox's Bazar displaced persons camp
Source: eClinicalMedicine. 2021 Nov 20;42:101205. doi: 10.1016/j.eclinm.2021.101205 (PMC8608865; doi:10.1016/j.eclinm.2021.101205)
Supplement: Supplementary file 5 [file mmc5.docx]

# Appendix 5: Sample Size Calculations

The sample sizes used (400 in the standard survey and 400 in the pictorial survey, split evenly between each of the two seasons) provided power to estimate the carer-reported diarrhoea rate in each arm (in both seasons combined) to a 95% confidence interval of approximately ±4 percentage points, assuming a point prevalence of 10%. The subsample providing stool samples (n=120) would provide precision to estimate the sensitivity and specificity values to approximately ±9 percentage points assuming values of 90%. Between the pictorial and standard questionnaire, using a 95% confidence interval will allow for a minimum detectable difference in reported diarrhoea rates of approximately 8 percentage points.
