# Supplementary material for: A comparison of traditional diarrhoea measurement methods with microbiological and biochemical indicators: A cross-sectional observational study in the Cox's Bazar displaced persons camp
Source: eClinicalMedicine. 2021 Nov 20;42:101205. doi: 10.1016/j.eclinm.2021.101205 (PMC8608865; doi:10.1016/j.eclinm.2021.101205)
Supplement: Supplementary file 6 [file mmc6.docx]

# Appendix 6: Weighting Scheme

As an illustrative example, there were 13 diarrhoea and 12 non-diarrhoea samples from the standard survey in the wet season. Using our estimated diarrhoea rate of 37.2% from the standard survey in the wet season, a random sample of 25 individuals asked the standard survey in the wet season would have 9.3 diarrhoea stools and 15.7 healthy stools. Diarrhoea stools from the standard survey in round one were thusly waited by 9.3/12 (0.715), and healthy stools by 15.7/12 (1.207). Weights were similarly obtained for the other survey types, seasons, and diarrhoea statuses:

|  |  | Diarrhoea | Healthy |
| --- | --- | --- | --- |
| Wet | Standard | 0.715 | 1.207 |
| Wet | Pictorial | 1.105 | 0.871 |
| Dry | Standard | 0.788 | 1.17 |
| Dry | Pictorial | 1.026 | 0.96 |
