# Supplementary material for: A comparison of traditional diarrhoea measurement methods with microbiological and biochemical indicators: A cross-sectional observational study in the Cox's Bazar displaced persons camp
Source: eClinicalMedicine. 2021 Nov 20;42:101205. doi: 10.1016/j.eclinm.2021.101205 (PMC8608865; doi:10.1016/j.eclinm.2021.101205)
Supplement: Supplementary file 7 [file mmc7.docx]

# Appendix 7: Demographics and Selected Diarrhoea Risk Factors

|  | **Dry Season** | | | | **Wet Season** | | | |
| --- | --- | --- | --- | --- | --- | --- | --- | --- |
|  | **Standard**  **(No Stool)** | **Standard**  **(Stool)** | **Pictorial**  **(No Stool)** | **Pictorial (Stool)** | **Standard**  **(No Stool)** | **Standard (Stool)** | **Pictorial**  **(No Stool)** | **Pictorial (Stool)** |
| **Total Respondents** | 147 | 25 | 145 | 31 | 163 | 35 | 140 | 31 |
| **Respondent Sex (Female)** | 91.8  [87.4, 96.3] | 100.0  [86.3, 100.0] | 91.03  [86.3, 95.7] | 100.0  [88.8, 100.0] | 77.3  [70.8, 83.8] | 85.7  [73.5, 97.9] | 75.0  [67.7, 82.3] | 93.9  [70.2, 97.6] |
| **Obtains Water from Tap** | 96.6  [93.6, 99.6] | 96.0  [87.7, 100.0] | 97.2  [94.5, 99.9] | 100.0  [88.8, 100.0] | 96.3  [93.4, 99.2] | 100.0  [90.0, 100.0] | 96.4  [93.3, 99.5] | 96.8  [90.2, 100.0] |
| **Stores Water in Closed Bucket** | 83.0  [76.8, 89.1] | 84.0  [68.5, 99.4] | 78.6  [71.9, 85.4] | 93.5  [84.4, 100] | 95.1  [91.7, 98.4] | 91.4  [81.7, 100.0] | 97.9  [95.4, 100.0] | 93.5  [84.3, 100.0] |
| **Does Something to Treat Water** | 25.2  [18.1, 32.3] | 12.0  [0, 25.7] | 21.4  [14.6, 28.1] | 38.7  [20.5, 56.9] | 68.1  [60.1, 75.3] | 65.7  [49.2, 82.3] | 71.4  [63.8, 79.0] | 64.5  [46.7, 82.3] |
| **Washes Hands with Water and Soap** | 75.5  [68.5, 82.5] | 88.0  [74.3, 100.0] | 79.3  [72.6, 86.0] | 80.6  [65.9, 95.4] | 96.9  [94.3, 99.6] | 94.3  [86.2, 100.0] | 97.1  [94.3, 99.9] | 100.0  [88.8, 100.0] |

Table 11 Caption: Percentages and 95% confidence intervals of key demographic and selected diarrhoea risk factors between seasons, arms, and stool collection status: *%[95%CI]*
