# Supplementary material for: A comparison of traditional diarrhoea measurement methods with microbiological and biochemical indicators: A cross-sectional observational study in the Cox's Bazar displaced persons camp
Source: eClinicalMedicine. 2021 Nov 20;42:101205. doi: 10.1016/j.eclinm.2021.101205 (PMC8608865; doi:10.1016/j.eclinm.2021.101205)
Supplement: Supplementary file 8 [file mmc8.docx]

# Appendix 8

We assume that in each of the major WASH trials the intervention is aiming to affect the prevalence of enteric infection and we measure self-reported diarrhoea instead. We also assume that there is a sensitivity and specificity as described in this paper. For the moment we assume that these are known and conduct a quick sensitivity analysis to examine how the point estimate of the relative risk from recent major WASH trials changes with different values of sensitivity and specificity. We examine only the point estimate as the standard errors reported in the papers reflect clustering, which we cannot take into account with only the reported case counts.

## Statistical model

We just assume a simple unadjusted log-binomial model to estimate relative risks. There is a baseline probability of experiencing enteric infection $p_{0}$ and the relative risk associated with the intervention $RR=\exp\left( \rho\right)$. We also have the sensitivity ($Se$), which is the probability someone with enteric infection reports diarrhoea, and the specificity ($Sp$), which is the probability someone without enteric infection does not report diarrhoea. Each trial records for arm $k=1$ (intervention) and arm $k=0$ (control) the sample size $n_{k}$ and the numbers reporting diarrhoea $z_{k}$. We assume both of these are binomially distributed:

$$z_{0}\sim Binomial\left( n_{0},\left( 1-Sp \right)+\left( Se+Sp-1 \right)p_{0} \right)$$

$$z_{1}\sim Binomial\left( n_{1},\left( 1-Sp \right)+\left( Se+Sp-1 \right)p_{0}exp(\rho) \right)$$

The likelihood for the data is

$$L\left( z_{1},z_{0} \right)=\binom{n_{1}}{z_{1}}\binom{n_{0}}{z_{0}}\left[ \left( 1-Sp \right)+\left( Se+Sp-1 \right)p_{0} \right]^{z_{0}}\left[ 1-\left( 1-Sp \right)-\left( Se+Sp-1 \right)p_{0} \right]^{n_{0}-z_{0}}\left[ \left( 1-Sp \right)+\left( Se+Sp-1 \right)p_{0}\exp\left( \rho\right) \right]^{z_{1}}\left[ 1-\left( 1-Sp \right)-\left( Se+Sp-1 \right)p_{0}\exp\left( \rho\right) \right]^{n_{1}-z_{1}}$$

We can then obtain the maximum likelihood estimate of $\exp\left( \rho\right)$ given the data reported by the trials. Note that values of 1-Specificity greater than the lowest reported prevalence do not typically result in meaningful estimates as there is not usually a combination of sensitivity, prevalence, and relative risk that could have reasonable generated the data. Also note that for relative risk (prevalence ratio) outcomes, specificity is the most important factor as when $Sp=1$ any false negatives cancel out:

$$E\left( \frac{\frac{z_{1}}{n_{1}}}{\frac{z_{0}}{n_{0}}} \right)=\frac{\left( 1-Sp \right)+\left( Se+Sp-1 \right)p_{0}exp(\rho)}{\left( 1-Sp \right)+\left( Se+Sp-1 \right)p_{0}}$$

*Using Luby 2018 WASH Benefits Bangladesh as an Example*

For the comprehensive intervention encompassing nutrition, and all WASH components, Benefits reports $n_{0}=3,517$, $z_{0}=200$, $n_{1}=1,861$, and $z_{1}=65$. The Table shows the MLE point estimates (the top row indicates the crude treatment effect for the intervention reported in the paper).

| **Sensitivity** | **Specificity** | **Relative risk** |
| --- | --- | --- |
| 1 | 1 | 0.61 |
| 1 | 0.99 | 0.53 |
| 1 | 0.98 | 0.41 |
| 1 | 0.97 | 0.18 |
| 0.9 | 1 | 0.61 |
| 0.9 | 0.97 | 0.18 |
| 0.8 | 1 | 0.61 |
| 0.8 | 0.97 | 0.18 |


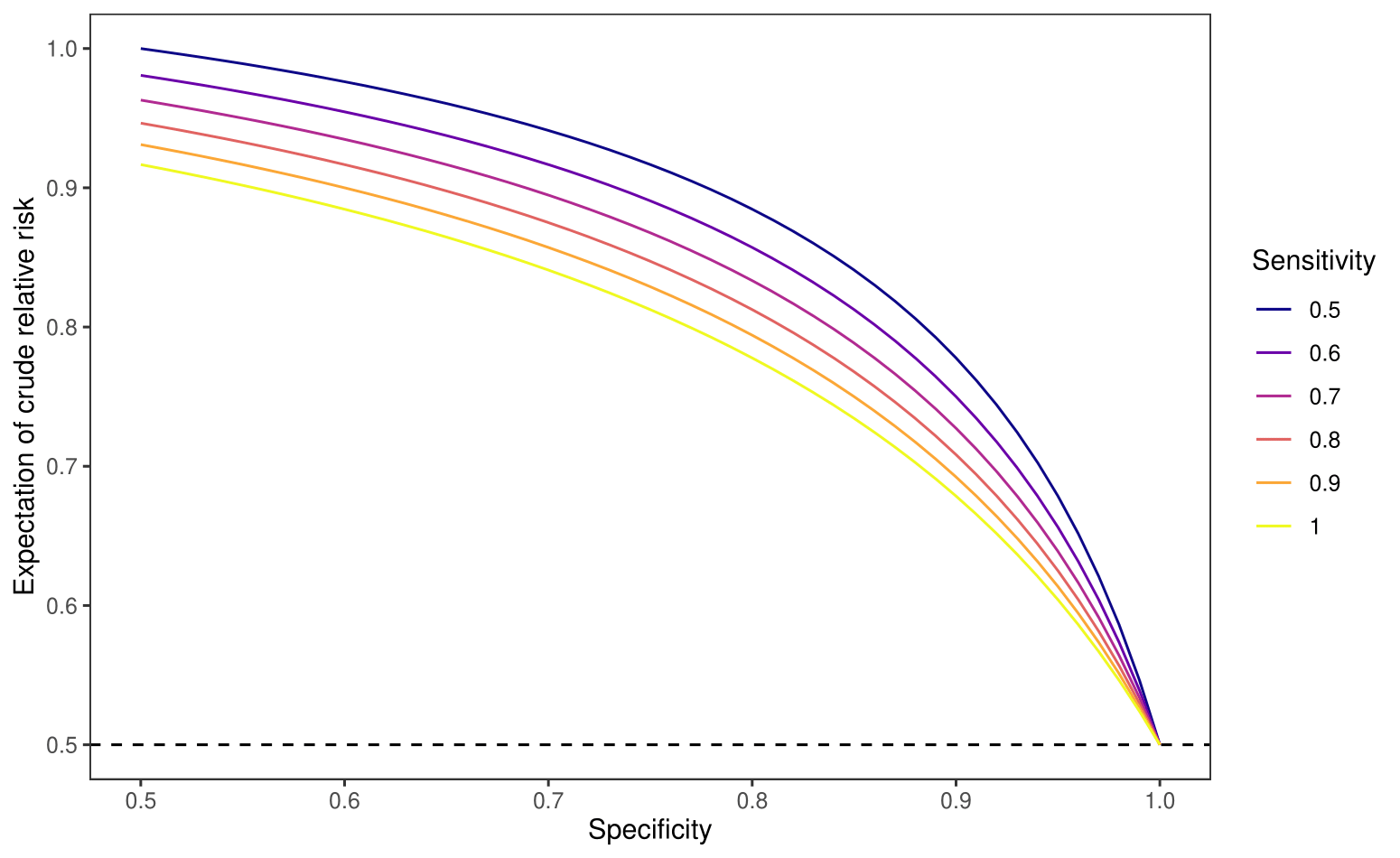


Relationship between outcome classification error and the expected value of the crude relative risk estimator. The prevalence of pathogen presence in the experimental and control groups are 20% and 10%, respectively, so the true relative risk is 0.5. Sensitivity = probability someone with infection reports diarrhoea, specificity = probability someone without carriage does not report diarrhoea
